# Methanolic leaf exctract of *Otostegia integrifolia* Benth reduces blood glucose levels in diabetic, glucose loaded and normal rodents

**DOI:** 10.1186/s12906-015-0535-5

**Published:** 2015-02-07

**Authors:** Zewdneh Shewamene, Mohammedberhan Abdelwuhab, Zewdu Birhanu

**Affiliations:** Department of Pharmacology, College of Medicine and Health Sciences, University of Gondar, P.O.Box 196, Gondar, Ethiopia

**Keywords:** Diabetes mellitus, *Otostegia integrifolia*, Streptozotoicn, Mice, Rats, Ethiopia

## Abstract

**Background:**

Although the leaves of *Otostegia integrifolia* traditionally claimed in Ethiopian folklore medicine for management of diabetes mellitus, it has not been validated. The aim of this study was therefore to investigate the antidiabetic activity of *Otostegia integrifolia* in rodents.

**Methods:**

Male rats or mice were randomly divided into five groups for diabetic, hypoglycemic and glucose tolerance test (OGTT) studies. In all models, group I received Tween 80, 2% v/v, Group II (GL5) treated with 5 mg/kg of glibenclamide and the remaining group: III, IV and V were given the plant extract at doses of 100 mg/kg 200 mg/kg and 400 mg/kg respectively. Blood glucose levels (BGL) were measured by collecting blood samples at different time points. Data was analyzed using one way ANOVA followed by Dunnet’s post hoc test to carry out between and within group comparisons. P < 0.05 was considered as statistically significant.

**Results:**

Inter-group analysis revealed that *O. integrifolia* at 100 mg/kg and 200 mg/kg reduced the 4^th^ hour fasting blood BGL significantly (p < 0.001) compared to the control group. The intra-group analysis result has shown *O. integrifolia* at 200 mg/kg produced a significant (p < 0.05) reduction in BGL at the 1^st^, 2^nd^, 3rd and 4th hours of post treatment compared to their respective initial levels. Moreover, in the hypoglycemic and OGTT models, *O. integrifolia* extract at 200 mg/kg, has shown a significant reduction in blood glucose levels compared to negative controls and across all time points.

**Conclusion:**

The crude extract of *O. integrifolia* has shown significant antidiabetic, hypoglyceamic and oral glucose tolerating effects. The effective dose of the extract was found to be 200 mg/kg in time dependent manner.

## Background

The global prevalence of diabetes mellitus (DM) for 2010 was 6.4%, affecting 285 million adults aged between 20–79 years and this figure will rise to a prevalence of 7.1% with 439 million diabetic adults by the year 2030 [1]. The goal of DM treatment is primarily to secure a quality of life and extend lifespan comparable to those of healthy people [2]. The use of drugs in management of DM however, is limited by the fact that they have adverse effects and higher secondary failure rates [3].

Numerous traditional medicinal plants have been used to treat patients with diabetes mellitus in various parts of the world especially in developing countries [4,5]. A large number of these plants or their preparation have been evaluated and confirmed to have hypoglyceamic effects in animal models [6,7] and some in human beings [8,9]. Some of antidiabetic plants have been experimentally evaluated and the active principles were isolated [4,7,10]. Most of these plants contain glycosides, alkaloids, terpenoids, flavonoids, polysaccharides, and saponins, which are frequently implicated to having antidiabetic effect. The practice of using plants for management of diabetes is also documented in Ethiopian traditional medicine system [11,12].

The genus Otostegia (Lamiaceae) comprises about 15 species. Five species of this genus have been reported to occur in flora of Ethiopia and Eritrea with various medicinal applications [13]. There are more than 65 new and novel compounds that have been isolated from this genus. Particularly, compounds from O.integrifolia, O.perisca and O.fruticosa were pharmacologically important. Naturally, the species of this genus traditionally been used as an ophthalmia, mosquito repellency, antimicrobial, antihyperglyceamic and antioxidant activity [14]. So far, *O. perisca* is well studied and documented as it possesses a good antidiabetic and antioxidant activity in animal models among the genus Otostegia [15-19].

In Ethiopia, *O.integrifolia* has been used traditionally for treatment of DM by the community documented in literatures [11,20] without any scientific proof for safety and efficacy. Althoug there is no previous investigation conducted in animals; several studies suggest that *O. integrifolia* is a good natural anti-oxidant that can be used as health-promoting agent for various disorders including DM [21]. The fact that hyperglycemia can be induced by generation of free radicals leading to the disruption of cellular functions, anti-oxidant agents are options in management of chronic disease like DM [22]. Moreover, effectiveness of *O.persica* in management of DM could indicate that *O.integrifolia* (in the same genus) may also be a potential antidiabetic agent that has to be investigated. Besides, bioactive constitutes with potential antihyperglyceamic and antioxidant activities were also isolated from *O.integrifolia* [14,23].

Thus, investigating the efficacy of this plant in animal model for the first time could give valuable information in this regard so that the society at large could benefit from the use of the plant. The results of pharmacological screening of claimed plant could have a contribution in the discovery of new medicinal plants with better antidiabetic activity. Besides, such study could deliver a baseline data for those researchers who are engaged to search medicinal plants with antidiabetic activity. In light of this, the present study was conducted to investigate the antidiabetic role of *O.integrifolia* in rodents.

## Methods

### Drugs and chemicals

All chemicals and drugs used in this experiment were maintained to be standards. Streptozotocin (Chengdu Yuyang High-tech Developing Co., Ltd, China), glucose standard strip/kits (Sensocard, Germany), glucometer (GLAB, Germany), glibenclamide (Sanofi-Aventis, USA), tween-80 (BDH Laboratory supplies Ltd, England), methanol absolute, (ReAgent chemical services Ltd, UK), hydrochloric acid (BDH Ltd, England) sulphuric acid (Farm Italia Carrloerba, Italy), acetic anhydride (Techno Pharmchem, India), ferric chloride (FISHER Scientific Company, New Jersey), potassium ferrocyanide (BDH Ltd, England), ferric sulphate (BDH Ltd, England) were some of important drugs and chemical used in this study.

### Plant material

The plant material, *Otostegia integrifolia* had collected from “Ayimba” 20 km away from Gondar (North West Ethiopia) in November 2013. Taxonomic identification was done and a voucher specimen (ZS001) was deposited at the College Natural Sciences, Addis Ababa University.

### Extraction procedure

The plant material was thoroughly washed with distilled water to remove dirt and soil, and dried under shade and optimal ventilation. Then pulverized and the powdered plant material was extracted thrice with 80% methanol. The resultant hydro-alcoholic extract was dried under reduced pressure and kept in a refrigerator until use.

### Preliminary phytochemical screening

The 80% methanolic leaf extract of *Otostegia integrifolia* was screened for the presence of alkaloids, flavonoids, polyphenols, tannins, saponins, steroidal compounds and reducing sugars according to established testing protocols [24-26].

### Experimental animals

Healthy Male Swiss albino mice (weighing 20–30 g and age of 8–12 weeks) and Wistar rats (weighing 150–300 g and age of 3 months) were obtained from the animal house of Gondar University and Addis Ababa University respectively. Animals were housed in polypropylene cages, maintained under standard condition (12 h light and 12 h dark cycle; 25-30°C) and allowed free access to standard pellet diet and water *ad libtum*. After randomized grouping and before initiation of the experiment, the animals were acclimatized to the laboratory conditions at the School of pharmacy, University of Gondar. All procedures complied according to The Guide for the Care and Use of Laboratory Animals [27] and approved by the Institutional Review Board of University of Gondar.

### Grouping and dosing of animals

Male animals were selected for the study based on previous study that demonstrated a better percent induction of diabetes using streptozotocin [12]. In all cases, group I was received 2% Tween, 2% v/v 80 (TW80); Group II received 5 mg/kg of glibenclamide (GL5); Group III, IV and V were treated with the extract at doses of 100 mg/kg (OI100), 200 mg/kg (OI200), and 400 mg/kg (OI400). The doses were selected from previous similar studies based on acute toxicity tests [24]. The extract was administered once only in all models.

### Hypoglycemic activity in normal mice

Mice were fasted for 4–6 h, but water was allowed *ad libitum*. Then, the mice were randomly divided into five different groups (6 per group). Group I was received 2% Tween, Group II received 5 mg/kg of glibenclamide, and Group III, IV and V were treated with the extract at doses of 100 mg/kg, 200 mg/kg, and 400 mg/kg. The animals were treated according to their respective grouping as mentioned above. Using aseptic conditions, blood sample was then collected from tails of the animals to determine blood glucose levels (BGL) at 0, 1, 2, 3 and 4 h post-treatment and the average BGL value was taken after triplicate measurements.

### Oral glucose tolerance test in rats

For the oral glucose tolerance tests (OGTT), rats were used since previous studies reported that rats are preferable instead of mice [12,28]. Rats were fasted overnight for 12–14 h and assigned randomly into 5 groups (n = 6/group). The rats were then orally treated according to their respective grouping. Thirty min after treatment, all of the rats were loaded with 2 g/kg glucose solution. Blood samples were collected from the tails of the animals to determine BGL as described above immediately prior to treatment and after treatment at 30, 60 and 120 min of glucose challenge.

### Induction of diabetes

Streptozotoin was dissolved in 0.1 M citrate buffer and PH was adjusted using HCl drops at 4.5. Then the solution was administered intraperitonially at 150 mg/kg dose to mice that was fasted for 4–6 h prior to administration. Seventy-two hours later, animals were screened for diabetes. Mice which showed blood glucose level > 200 mg/dL were included in the study [12].

### Effects of the extract on diabetic mice

Streptozotocin induced diabetic mice were randomly divided into five groups (n = 6/group) and treated with the extract according to their respective groups. Blood samples were collected from the tails of the animals to determine BGL at 0, 1, 2, 3 and 4 h post treatment.

### Statistical analysis

All data was expressed as mean ± SEM. Between and within group analysis were carried out using one way ANOVA followed by Dunnet’s post hoc test. The results were considered to be significant when the P-value was less than 0.05. For data processing SPSS data analysis soft ware Version 20.0 was used.

## Results

### Preliminary phytochemical screening

The 80% methanol leaf extract of *O. integrifolia* gave a greenish brown semi-solid product with a percentage yield of 19%. Preliminary phytochemical screening indicated that the extract contains phenolic compounds, saponins, reducing sugars and flavonoids. Alkaloids, tannins and steroidal compounds were absent.

### Effects on streptozotocin induced diabetic mice

As revealed in Table 1, between and within group analysis were performed to see BGL differences across the various groups and time points respectively. The inter-group analysis indicated that no detectable changes were noted between the initial, 1 h and 2 h BGL of all groups. GL5 has shown to reduce the BGL significantly (58.7%; p < 0.001) compared to the negative control at 3 h. Similarly, GL5, OI 100 and OI 200 were able to lower the 4^th^ hour fasting BGL significantly (p < 0.001) compared to the control group. However, glibenclamide induced BGL reduction was more pronounced (63%), than both OI 100 and OI 200 (49%, 57.6%) respectively even if this difference is not statistically significant. There was no apparently significant blood glucose change in OI 400 compared to the control group in all time points. Moreover, fasting BGL did not significantly varied between groups treated with the extract at all time points though 200 mg was found more effective than the other two doses.

**Table 1:** **Antidiabetic effects** ***O*** **.** ***interifolia*** **80% methanolic leaf extract on fasting blood glucose levels in streptozotocin induced mice**

**Blood glucose levels (mg/dl)**					
**Groups**	**0 h**	**1 h**	**2 h**	**3 h**	**4 h**
TW80	347.61 ± 41.81	362.83 ± 43.81	402.55 ± 22.67	402.22 ± 26.51	375.50 ± 23.71
GL5	337.22 ± 33.32	304.94 ± 36.03	217.33 ± 17.10^b2^	166.16 ± 13.27^a3b3c2^	139.38 ± 12.00^a3b3c3^
OI 100	424.58 ± 59.47	407.66 ± 63.11	350.08 ± 67.63	279.08 ± 60.26	191.83 ± 45.53^a3^
OI 200	396.25 ± 57.88	364.08 ± 60.11	318.41 ± 60.98	243.33 ± 63.24	159.83 ± 71.10^a3b1^
OI 400	386.66 ± 57.13	312.00 ± 54.82	270.33 ± 61.98	228.41 ± 51.48	207.00 ± 44.25

Values are mean ± SEM. n = 6; ^a^compared to TW80; ^b^compared to 0 h ^c^compared to 1 h. ^1^p < 0.05; ^2^p < 0.01; ^3^p < 0.001. (TW80, control that received Tween80; GL5, positive control received glibenclamide 5 mg/kg: OI 100, extract 100 mg/kg; OI 200, extract 200 mg/kg; OI 400, extract 400 mg/kg).

Within group comparison indicated that significant BGL reduction was not observed in TW80, OI 100 and OI 400 groups along with all time points. However, percent reduction in BGL was recorded as 55% and 46% at 4 h compared to 0 h in OI 100 and OI 400 groups respectively. Glibenclamide produced a significant BGL reduction (p < 0.01; p < 0.001) at 3^rd^ and 4^th^ hours respectively compared to the initial level. The extract at 200 mg was able to decrease the BGL significantly (60%; p < 0.05) on the 4^th^ hour compared to initial value (Table 1).

### Hypoglycemic effect in normal mice

The inter-group analysis indicated that no detectable changes were noted between the initial BGL of all groups. Similarly, BGL did not affected significantly after 30 minutes of treatment in all groups. GL5 group has shown a significant (50.7%; p < 0.00 and 54%, p < 0.001) reduction in BGL compared to the TW80 at 1 h and 2 h respectively. GL5 also reduced BGL significantly compared to OI 100 and OI 400 (P < 0.001) at both 1 h and 2 h. Treatment with OI 200 mg has lowered the fasting BGL significantly (p < 0.05) compared to control, OI 100 mg and OI 400 mg at 1 h. A significant BGL difference between GL5 and OI 200 was not observed at all time points which has meant that the extract at 200 mg/kg is comparably effective with glibenclamide but not equieffective (Table 2). Within group analysis revealed that TW80 treated animals did not show significant reduction in BGL across all time points compared to the initial level. Similarly, OI400 failed to show significant hypoglyceamic effect at all time point. By contrast, OI100 and OI200 produced a significant (p < 0.05) reduction in BGL at the 1^st^, 2^nd^, 3rd and 4th hours of post treatment compared to their respective initial levels. Likewise, GL5 brought about significant reduction at all time points compared to the initial BGL (Table 2).

**Table 2:** **Hypoglycemic effects** ***O*** **.** ***interifolia*** **80% methanolic leaf extract on fasting blood glucose levels in normoglycemic mice**

**Blood glucose levels (mg/dl)**					
**Groups**	**0 h**	**1 h**	**2 h**	**3 h**	**4 h**
TW80	92.50 ± 1.05	87.66 ± 2.51	84.77 ± 3.85	83.11 ± 2.72	83.27 ± 4.26
GL5	90.83 ± 4.87	59.16 ± 3.41^a2b3c1d3e3^	61.38 ± 2.72^a2d1e3^	57.66 ± 3.12^a3d3e3f3^	48.50 ± 1.59^a3d3e3f3^
OI 100	89.66 ± 6.44	97.58 ± 3.81^e3^	78.50 ± 6.15^e3^	66.16 ± 2.85^a1d1e3^	57.66 ± 3.87^a3d3e3f3^
OI 200	94.75 ± 5.11	80.3 ± 3 7.09^e3^	64.08 ± 4.38^a1e3^	55.75 ± 2.65^a3d3e3f3^	49.50 ± 1.24^a3d3e3f3^
OI 400	99.58 ± 5.90	89.25 ± 6.25	82.66 ± 5.26	85.08 ± 6.00	84.75 ± 5.59

Values are mean ± SEM. n = 6; ^a^compared to TW80; ^b^compared to OI 100; ^c^compared to OI 200; ^d^compared to OI 400; ^e^compared to 0 h: ^f^compared to 1 h. ^1^p < 0.05; ^2^p < 0.01; ^3^p < 0.001. (TW80, control that received Tween80; GL5, positive control received glibenclamide 5 mg/kg: OI 100, extract 100 mg/kg; OI 200, extract 200 mg/kg; OI 400, extract 400 mg/kg).

### Effects on oral glucose tolerance test in rats

As show in Table 3, the initial fasting blood glucose levels (FBGL) of all groups showed no apparent difference compared with each other. All groups, however, showed significant increase (p < 0.001) in FBGL one hour following oral glucose challenge (or 30 min post extract treatment), confirming the induction of hyperglyceamia. The extract at 200 mg/kg has shown a significant blood glucose reduction (27%; p < 0.05) at the 1^st^ hour of treatment compared the control counterpart. In contrast, both 100 mg and 400 mg treated groups were not having significantly reduced blood glucose compared to the control.

**Table 3:** **The effects of** ***O.integriolia*** **80% methanolic extract on Oral Glucose Tolerance Test in rats**

**Blood glucose levels (mg/dl)**				
**Groups**	**0 h**	**0.5 h**	**1 h**	**2 h**
TW80	86.11 ± 1.10	166.77 ± 3.037	148.22 ± 4.78	135.72 ± 3.02
GL5	87.00 ± 1.94	150.27 ± 4.40	73.16 ± 2.49^a3b3c3 3d3^	62.22 ± 2.35^a3b2c3d3^
OI 100	91.25 ± 2.27	182.16 ± 11.53	149.50 ± 9.86 ^d3^	102.33 ± 4.01^a1d3 e3^
OI 200	92.08 ± 2.71	172.58 ± 19.03	108.83 ± 9.01^a1b1c1 d3^	85.33 ± 3.16^a3c3 d3^
OI 400	90.16 ± 1.70	183.25 ± 21.42	149.00 ± 14.32^e3^	157.91 ± 15.53^f1^

Values are mean ± SEM. n = 6; ^a^compared to TW80; ^b^compared to OI 100; ^c^compared to OI 400; ^d^compared to 30 min: ^e^compared to 30 min: ^f^compared to 1 h. ^1^p < 0.05; ^2^p < 0.01; ^3^p < 0.001. (TW80, control that received Tween80; GL5, positive control received glibenclamide 5 mg/kg: OI 100, extract 100 mg/kg; OI 200, extract 200 mg/kg; OI 400, extract 400 mg/kg).

As usual, glibenclamide treated group has shown significantly (p < 0.001) lower blood glucose at 1^st^ and 2^nd^ hours of treatment compared to TW80, OI100 and OI400 groups. However, the BGL lowering effect between GL5 and OI200 was not significantly different at all time points. Intra-group BGL was found to be significantly lowered in GL5, OI100 and OI200 at 1^st^ and 2^nd^ hours of treatment compared to the 30^th^ minutes glucose level (Table 3).

## Discussion

A recent study has estimated that up to 30% of patients with diabetes mellitus use complementary and alternative medicine [29]. Medicinal plants and its products continue to be an important therapeutic aid for alleviating the ailments of human kind [30-32]. Developing agents for management of DM that are devoid of adverse effects are still a challenge to the medical care system. This has led to an increased demand for agents known to have fewer side effects than the contemporary oral hypoglyceamic agents. Natural products with antihyperglycaemic activity are stated to have fewer side effects than synthetic oral hypoglyceamic agents [33]. Thus, the research on hypoglyceamic plants is increasing which might offer a natural key to a better clinical management of DM in the future.

Acute toxicity studies revealed the non-toxic nature of the hydroalcoholic extract of the leaves of O. *integrifolia*. There were no lethality or toxic reactions observed at the limit dose level until the end of the study period. The median lethal dose (LD50) of the plant extract is said to be greater than 5000 mg/kg, indicating a good safety margin [24].

Streptozotocin-induced hyperglycaemia has been described as a useful experimental model to study the activity of hypoglycaemic agents [34,35]. Streptozotocin exhibits a cytotoxic activity and causes the death of pancreatic β-cell by alkylation of DNA, leaving less active cells and thereby, attenuating insulin synthesis and release. Furthermore, it has been shown to be involved in the fragmentation of DNA by means of production of reactive oxygen species. Due to this it is commonly used to induce diabetes [36].

The current experiment was carried out based on high-dose streptozotocin induction protocol in mice forwarded by the Animal Models of Diabetic Complications Consortium [34]. To formulate uniform solutions, the extract was dissolved in 2% of tween-80 which allowed easy administration. Verma et al., (2010) used tween-80 as a suspending agent for similar study since the effect on BGL is stated to be negligible [37]. In line with this, the results from this study demonstrated that TW80 remained to produce insignificant change in BGL.

The result of the present study indicated that diabetic and normal mice of the negative control group demonstrated no apparent deference in BGL when the base line BGL was compared with the other intervals. Similarly, in OGTT there was no significant BGL difference between the peak hyperglycemic level and the next intervals (60 and 120 min) in the negative control group. However, there were significant reductions of BGL in diabetic, normal and glucose loaded groups in when *O.integrifolia* extract and the standard drug were administered, indicating that changes produced were attributed to treatment received. Therefore, the results of this study indicated that extract of *O. integrifolia* reduces blood glucose level in normal and diabetic mice as well as in glucose induced hyperglyceamic rats.

This study revealed that maximum antidiabetic, hypoglyceamic and oral glucose tolerance activities were observed with OI200. It is interesting to note that OI200 was capable of lowering hyperglyceamia level in streptozotocin-induced diabetic mice which was very close to the normal range, similar to the standard glibenclamide. The extract also brought the hyperglycemic state in OGTT down to BGL, which was similar with the baseline within 60 min as well as the standard drug. Thus, it is plausible to assume that the plant extract and glibenclamide might produce hypoglyceamic and antidiabetic activities by similar mechanisms. Extracts of other plants, including *Cassia italic* [38] and *Vinca rosea* [39] have been reported to have similar mode of action with that of glibenclamide, lending evidence to the earlier assertion.

It was observed that the extract exerted its action in a non-dose dependent fashion; particularly the higher dose produced less activity. OI400 did produce a delayed but significant antidiabetic, hypoglyceamia and glucose tolerance but not essentially significant unlike OI200. Indeed, such diminished hypoglyceamic responses at higher dose have been reported previously in plants such *Murraya koenigii* [40] and *Cynodon dactylon* [41]. Diminished effects of higher doses of the extract could result from the interference of constituents that may raise BGL as they get increased in concentration following an increase in dose. As the study was carried out on crude extract of *O. integrifolia* BGL reduction, probably, is the net effect of the interplay of various constituents of the extract. It is likely that higher doses may activate non- specifically both BGL lowering and rising mechanisms. It is reported that the presence of non-hypoglyceamic and/or antagonistic substances in the extract may diminish hypoglyceamic effect [42].

On the other hand, the lower dose (100 mg/kg) of the extract was ineffective in producing and effect similar to OI200 and it could be due to inability of the dose to overcome counter regulatory factors physiologically involved in response to hypoglyceamia such as glucagon, cortisol, adrenaline [43] or it is likely that it might have lesser concentration of the active principles to give rise to frank hypoglyceamia. Despite lack of statistical significance, the hypoglyceamic effect of this dose is very noticeable from the results observed. It was also shown that the activity of the extract increased with time. Significant antidiabetic action was at all time points, with maximal effect achieved at the 4^th^ h. This might mean that the active principles in the extracts take time to reach sufficient concentration at the target site. Sharma et al. (1996) reported that Aegle marmelos leaves extracts showed similar delay in maximum effect compared to the onset of action and forwarded similar explanation [44].

The plant extract showed relatively delayed antidiabetic onset of action than the standard drug. However, the doses of *O.integrifolia* extract were varied in onset of action. This could be due to the interference of non-hypoglyceamic actions, as it was the case for non-dose dependency, particularly in the higher dose. It could also be due to principles such as reducing sugars or other carbohydrate which might interfere with the onset and hypoglyceamic effect of the extract. Because, such phytochemicals with higher glycaemic index (such as reducing sugars) could give rise to free glucose after digestion and following absorption they may tend to rise BGL in the face of the hypoglyceamic actions by the active hypoglyceamic agents. And higher doses of the extract may result in higher concentrations of such agents. For instance, high molecular weight compounds such as proteins and kinins, which might cause the release of histamine, were stated to be associated with raise in blood glucose level before hypoglycemic action were observed [45-47]. Therefore, the discrepancies of hypoglycemic action of *O. integrifolia* extract could be due to reducing sugars or undetected components in the extract.

DM involves over production (excess hepatic glycogenolysis and gluconeogenesis) and decreased utilization of glucose by tissues underlying hyperglyceamia. Hyperglycemia induces the generation of free radicals leading to the disruption of cellular functions, oxidative damage to membranes and increased susceptibility to lipid peroxidation [43]. A glucose tolerance test is a measure of the body’s ability to utilize glucose. Interestingly, in OGTT the hydroalcolic extract showed significant reduction in blood glucose level from 60 min onwards except for the higher dose. This suggests that the extract enhanced glucose utilization which brought about a significantly decreased in BGL. This could be a potential advantage of the extracts in minimizing hyperglyceamia related complications of diabetes, if the extract continues to demonstrate similar action in chronic/repeated administration.

Preliminary phytochemical screening of the hydroalcoholic extract of *O.integrifolia* demonstrated the presence of saponins, flavonoids, and phenolic compounds, hypoglyceamic effects in other plants. Flavonoids have been shown to have a significant antidiabetic activity and are also known for their ability to regenerate pancreatic beta cells [45]. Phenolic constituents of plants have been reported to have a significant blood glucose lowering effect [46]. Thus, the good antidiabetic, hypoglyceamic and enhanced glucose utilization effect of the hydroalcoholic extract of *O.integrifolia* may be associated with the presence of these different secondary metabolites with possible synergistic effects.

## Conclusions

The crude extract of *O. integrifolia* has shown significant antidiabetic, hypoglyceamic and oral glucose tolerance improving effects. The effective dose of the extract was found to be 200 mg/kg. Moreover, it supports the traditional use of this plant in management of DM.
